# Racial and Ethnic Disparities in Clinical Trial Enrollment Among Women With Gynecologic Cancer

**DOI:** 10.1001/jamanetworkopen.2023.46494

**Published:** 2023-12-07

**Authors:** Wafa Khadraoui, Caitlin E. Meade, Floor J. Backes, Ashley S. Felix

**Affiliations:** 1 Department of Obstetrics and Gynecology, Division of Gynecologic Oncology, The Ohio State University Comprehensive Cancer Center, James Cancer Hospital and Solove Research Institute, Columbus; 2 Division of Epidemiology, College of Public Health, The Ohio State University, Columbus

## Abstract

**Importance:**

Racial and ethnic disparities in clinical trial enrollment are unjust and hinder development of new cancer treatments.

**Objective:**

To examine the association of race and ethnicity with clinical trial enrollment among women with endometrial, ovarian, or cervical cancer.

**Design, Setting, and Participants:**

This retrospective cohort study used data from the National Cancer Database, a hospital-based cancer registry, and the Surveillance, Epidemiology, and End Results Program (SEER), a population-based cancer registry. Population-based race and ethnicity–specific proportions for each cancer site were derived from SEER. Participants included women with an endometrial, ovarian, or cervical cancer diagnosed from 2004 to 2019. Analyses were performed from February 2 to June 14, 2023.

**Exposure:**

Race and ethnicity were categorized as American Indian/Alaska Native, Asian, Black, Hispanic (any race), Native Hawaiian/Pacific Islander, White, and other (not defined in the National Cancer Database).

**Main Outcomes and Measures:**

The primary outcomes were the odds of clinical trial enrollment and representation in clinical trials compared with the US population. Multivariable-adjusted logistic regression was used to estimate odds ratios (ORs) and 95% CIs for associations of race and ethnicity with clinical trial enrollment within the National Cancer Database sample. Participation-to-prevalence ratios (PPRs) according to diagnosis period (2004-2011 vs 2012-2019) were calculated by dividing the race and ethnicity–specific percentage of clinical trial participants in the study sample by the percentage of racial and ethnic groups in SEER.

**Results:**

Among 562 592 patients with gynecologic cancer (mean [SD] age at diagnosis, 62.9 [11.3] years), 1903 were American Indian/Alaska Native, 18 680 were Asian, 56 421 were Black, 38 145 were Hispanic, 1453 were Native Hawaiian/Pacific Islander, 442 869 were White, and 3121 were other race and ethnicity. Only 548 (<1%) were enrolled in clinical trials. Compared with White women, clinical trial enrollment was lower for Asian (OR, 0.44; 95% CI, 0.25-0.78), Black (OR, 0.70; 95% CI, 0.50-0.99), and Hispanic (OR, 0.53; 95% CI, 0.33-0.83) women. Compared with the US population, White women were adequately or overrepresented for all cancer types (PPRs ≥1.1), Black women were adequately or overrepresented for endometrial and cervical cancers (PPRs ≥1.1) but underrepresented for ovarian cancer (PPR ≤0.6), and Asian and Hispanic women were underrepresented among all 3 cancer types (PPRs ≤0.6).

**Conclusions and Relevance:**

In this cohort of patients with gynecologic cancer, clinical trial enrollment was lower among certain minoritized racial and ethnic groups. Continued efforts are needed to address disparate clinical trial enrollment among underrepresented groups.

## Key Points

**Question:**

Are there racial and ethnic disparities in clinical trial enrollment among women with gynecologic cancer?

**Findings:**

In this cohort study of 562 592 women with endometrial, ovarian, or cervical cancer, the odds of clinical trial enrollment were lower among Asian, Black, and Hispanic women compared with White women. Comparisons with the US population demonstrated overrepresentation among White women for all cancer sites, underrepresentation among Asian and Hispanic women for all cancer sites, and varied patterns for Black women depending on cancer site.

**Meaning:**

These findings suggest that efforts to engage women with gynecologic cancer who are from minoritized racial and ethnic groups are needed to increase their representation in clinical trials.

## Teaser

This cohort study examines the association of race and ethnicity with clinical trial enrollment among women with endometrial, ovarian, or cervical cancer.

## Introduction

Health care disparities exist within all scopes of medicine and occur along various dimensions, including race and ethnicity, socioeconomic status, geography, and language. Racial and ethnic inequities in gynecologic oncology treatment and outcomes are well-established and deeply entrenched in the social determinants of health,^1^ prompting calls to address these gaps in care.^2^ Clinical trials, defined as research in which humans are prospectively assigned to 1 or more interventions for the evaluation of health-related effects,^3^ are essential for ensuring validity, generalizability, and equity of care, as well as advancing medical knowledge. Recent reports^4,5^ suggest that between 6% and 8% of the US adult population with cancer participates in clinical trials, with lower representation of patients from minoritized racial and ethnic groups. Structural barriers (eg, lack of clinical trials in regions with a higher density of minoritized patients) and clinical factors (eg, narrow eligibility criteria that disproportionately affect underrepresented populations) have resulted in lower clinical trial enrollment of racial and ethnic minoritized groups,^4^ with evidence that this contributes to poorer survival.^6,7,8,9^

In a recent review, Barry and colleagues^10^ outlined the extent of racial disparities in clinical trial enrollment of patients with gynecologic cancer. Most of the reviewed studies compared observed enrollment in clinical trials identified through ClinicalTrials.gov with expected enrollment derived from population-based, age-adjusted incidence rates. Collectively, women from minoritized racial and ethnic groups were underrepresented in these trials, whereas White women were more likely to be overrepresented across gynecologic cancer types. This work is an important starting point for describing racial and ethnic disparities in clinical trial enrollment of patients with gynecologic cancer; however, studies with an internal comparison group with adjustment for potential confounders are needed to fully understand the complex picture of clinical trial enrollment. Moreover, these studies highlight the need for data sets that include large numbers of women from underrepresented groups. As such, we examined associations of race and ethnicity with clinical trial enrollment among women with gynecologic cancer using the National Cancer Database (NCDB). In addition, we present participation-to-prevalence ratios (PPRs) according to period of diagnosis to evaluate trends in the representation status of underrepresented groups in gynecologic cancer trials.

## Methods

### Data Source

The 2020 Participant User File was obtained from the hospital-based NCDB,^11^ a cancer registry capturing 70% of cancers diagnosed in the US. Data include sociodemographic characteristics, tumor characteristics, treatment facility attributes, treatment, and survival outcomes abstracted from patient medical records by Certified Tumor Registrars.^12^ Data submitted to NCDB undergo rigorous quality checks according to American College of Surgeons standards. This study was exempt from the Ohio State University institutional review board and the need for informed consent because the data were anonymous and publicly available, in accordance with 45 CFR §46. We followed the Strengthening the Reporting of Observational Studies in Epidemiology (STROBE) reporting guidelines for cohort studies.^13^

### Study Sample

We used *International Classification of Disease for Oncology, Third Edition* primary site codes to identify women (aged ≥18 years) with 1 of the following gynecologic cancers diagnosed between 2004 and 2019: endometrial (C54.0-C54.9 and C55.9), cervical (C53.0, C53.1, C53.8, and C53.9), ovarian (C56.9), fallopian tube (C57.0), peritoneal (C48.1 and C48.2), and retroperitoneal (C48.0).^11,12^ We restricted the sample to common histologic types (eTable 1 in Supplement 1), resulting in an initial sample size of 1 018 044 women. We excluded women for the following reasons: unknown race (51 384 women), unknown facility location or type (60 441 women), unknown income or education level (105 963 women), unknown clinical trial enrollment (71 women), noninvasive or unknown cancer stage (111 827 women), unknown radiation treatment (17 330 women), unknown chemotherapy (8019 women), and unknown follow-up time (341 women). Additional cancer site-specific exclusions are detailed in the eAppendix in Supplement 1. Following exclusions, ovarian, peritoneum, retroperitoneum, and fallopian tube cancers were grouped together for analysis.

### Clinical Trial Enrollment

We categorized women as enrolled in a clinical trial when response observations were enrolled in an institutional (code 2) or double-blind clinical trial (code 3). Categories of no trial (code 0), other (code 1), other–unproven (code 6), or refused trial (code 7) were categorized as no clinical trial enrollment.^14^

### Covariates

Race and ethnicity were available as self-reported variables coded by the NCDB. We cross-classified race (American Indian/Alaska Native, Asian, Black, Native Hawaiian/Pacific Islander, White, and other) and ethnicity (Hispanic vs non-Hispanic) to produce the following categories: non-Hispanic Asian (hereafter referred to as Asian), non-Hispanic American Indian/Alaska Native (hereafter referred to as American Indian/Alaska Native), non-Hispanic Black (hereafter referred to as Black), Hispanic ethnicity of any race, non-Hispanic Native Hawaiian/Pacific Islander (hereafter referred to as Native Hawaiian/Pacific Islander), non-Hispanic White (hereafter referred to as White), and non-Hispanic other (hereafter referred to as *other*). The NCDB does not specify what groups are included in “other race.” Detailed information on the categories of race that compose the 6 overarching groups is provided in the eAppendix in Supplement 1.

Additional covariates included age at diagnosis (continuous), Charlson-Deyo comorbidity score (0, 1, or ≥2), health insurance (none, private, Medicaid, Medicare, other government), area-level annual income (<$46 277, $46 277-$57 856, $57 857-$74 062, and ≥$74,063), area-level educational attainment (measure of the percentage of adults who did not graduate from high school; ≥15.3%, 9.1%-15.2%, 5.0%-9.0%, and <5.0%), metropolitan status (large metropolitan county [population >1 million], medium metropolitan county [population 250 000-1 million], small metropolitan county [population <250,000], urban, and rural), facility location (Northeast, Midwest, Mountain, Pacific, and South), facility type (community cancer, comprehensive community cancer, academic or research, and integrated network cancer), surgery (yes or no), chemotherapy (yes or no), radiation (yes or no), cancer stage (I, II, III, and IV), and tumor grade (1, 2, or 3; applicable for uterine and ovarian endometrioid and ovarian serous only). Additional details regarding area-level income, area-level education, metropolitan status, and cancer stage are provided in the eAppendix in Supplement 1.

### Statistical Analysis

In the NCDB sample, we used multivariable logistic regression to estimate adjusted odds ratios (ORs) and 95% CIs for associations of race and ethnicity with clinical trial enrollment. Factors included as covariates comprised patient, facility, tumor, and treatment characteristics that have been identified as factors related to clinical trial enrollment among patients with cancer^4^ and were available in NCDB.

To evaluate the racial and ethnic composition of patients with gynecologic cancer enrolled in clinical trials (in the NCDB) relative to the racial distribution in the overall cancer-specific population, we calculated the PPR according to period of diagnosis (2004-2011 vs 2012-2019).^15^ The PPR was calculated by dividing the race-specific percentage of clinical trial participants in the study sample (eg, percentage of NCDB patients with endometrial cancer enrolled in clinical trials who are Black) by the percentage of racial and ethnic groups in the US patient population (eg, percentage of US patients with endometrial cancer who are Black) according to cancer site. We used the Surveillance, Epidemiology, and End Results *Stat program to derive population-based race and ethnicity frequencies for each cancer site. We omitted calculations for American Indian/Alaska Native, Native Hawaiian/Pacific Islander, and other women owing to low numbers. Stratification of the PPR by diagnosis period (2004-2011 vs 2012-2019) was done to qualitatively assess clinical trial enrollment over time. We evaluated only 2 time periods to reduce the potential for small numbers. PPRs less than 0.8 can be interpreted as underrepresentation in clinical trials, PPRs of 0.8 to 1.2 indicate adequate representation in clinical trials, and PPRs greater than 1.2 indicate overrepresentation.^15^ Additional methodological details are presented in the eAppendix in Supplement 1.

Statistical analyses were performed using Surveillance, Epidemiology, and End Results *Stat software version 8.4.1.1 (National Cancer Institute) and SAS statistical software version 9.4 (SAS Institute). All *P* values were 2 sided, with statistical significance set at *P* < .05. Analyses were performed from February 2 to June 14, 2023.

## Results

Among 562 592 women included (mean [SD] age at diagnosis, 62.9 [11.3] years), 1903 were American Indian/Alaska Native, 18 680 were Asian, 56 421 were Black, 38 145 were Hispanic, 1453 were Native Hawaiian/Pacific Islander, 442 869 were White, and 3121 were other race and ethnicity. Only 548 women (<1%) were enrolled in a clinical trial. In a multivariable-adjusted model, compared with White women, clinical trial enrollment was lower among Asian (OR, 0.44; 95% CI, 0.25-0.78), Black (OR, 0.70; 95% CI, 0.50-0.99), and Hispanic (OR, 0.53; 95% CI, 0.33-0.83) women but not significantly different for American Indian/Alaska Native (OR, 1.37; 95% CI, 0.43-4.36), Native Hawaiian/Pacific Islander (OR, 0.86; 95% CI, 0.12-6.16), or other race (OR, 0.48; 95% CI, 0.12-1.92) women (Table).

**Table.** Associations of Epidemiologic, Facility, Tumor, and Treatment Characteristics With Clinical Trial Enrollment Among Women With Gynecologic Cancer

Characteristic	Participants, No. (%) (N = 562 592)		Multivariable-adjusted OR (95% CI)^a^	*P* value
	Not enrolled in a clinical trial (n = 562 044)	Enrolled in a clinical trial (n = 548)		
Race				
American Indian/Alaska Native	1900 (0.3)	3 (0.6)	1.37 (0.43-4.36)	.006
Asian	18 668 (3.3)	12 (2.2)	0.44 (0.25-0.78)	
Black	56 382 (10.0)	39 (7.1)	0.70 (0.50-0.99)	
Hispanic	38 124 (6.8)	21 (3.8)	0.53 (0.33-0.83)	
Native Hawaiian/Pacific Islander	1452 (0.3)	1 (0.2)	0.86 (0.12-6.16)	
White	442 399 (78.7)	470 (85.8)	1 [Reference]	
Other^b^	3119 (0.6)	2 (0.4)	0.48 (0.12-1.92)	
Age, mean (SD), y	62.9 (11.3)	60.4 (9.7)	0.89 (0.85-0.94)	<.001
Charlson-Deyo score				
0	431 202 (76.7)	455 (83.0)	1 [Reference]	.09
1	99 097 (17.6)	78 (14.2)	1.01 (0.79-1.28)	
≥2	31 745 (5.7)	15 (2.7)	0.56 (0.34-0.95)	
Insurance status				
No insurance	21 004 (3.7)	16 (2.9)	1 [Reference]	.01
Private insurance	257 547 (45.8)	319 (58.2)	1.27 (0.76-2.12)	
Medicaid	41 017 (7.3)	31 (5.7)	0.77 (0.42-1.41)	
Medicare	227 861 (40.5)	173 (31.6)	1.00 (0.57-1.74)	
Other government	5559 (1.0)	8 (1.5)	1.47 (0.62-3.49)	
Unknown	9056 (1.6)	1 (0.2)	0.13 (0.02-1.01)	
Area-level annual income, $				
Quartile 1: <46 277	95 079 (16.9)	68 (12.4)	1 [Reference]	.03
Quartile 2: 46 277 to 57 856	121 797 (21.7)	108 (19.7)	0.95 (0.69-1.31)	
Quartile 3: <57 857 to 74 062	134 864 (24.0)	113 (20.6)	0.69 (0.49-0.98)	
Quartile 4: ≥74 063	210 304 (37.4)	259 (47.3)	0.65 (0.45-0.94)	
Area-level education, % of residents without high school diploma				
Quartile 1: <5.0	121 232 (21.6)	176 (32.1)	1 [Reference]	<.001
Quartile 2: 5.0 to 9.0	162 688 (29.0)	179 (32.7)	0.83 (0.66-1.03)	
Quartile 3: 9.1 to 15.2	157 292 (28.0)	126 (23.0)	0.59 (0.45-0.77)	
Quartile 4: ≥15.3	120 832 (21.5)	67 (12.2)	0.41 (0.28-0.59)	
Metropolitan status				
Large metropolitan county (population >1 million)	296 837 (52.8)	331 (60.4)	1 [Reference]	.03
Medium metropolitan county (population 250 000-1 million)	115 544 (20.6)	93 (17.0)	0.73 (0.58-0.93)	
Small metropolitan county (population <250 000)	49 091 (8.7)	39 (7.1)	0.70 (0.49-0.99)	
Urban	71 880 (12.8)	54 (9.9)	0.70 (0.51-0.96)	
Rural	8883 (1.6)	9 (1.6)	1.10 (0.55-2.16)	
Unknown	19 809 (3.5)	22 (4.0)	0.67 (0.43-1.04)	
Facility location				
Northeast	125 477 (22.3)	179 (32.7)	1 [Reference]	.007
South	198 948 (35.4)	161 (29.4)	0.72 (0.57-0.90)	
Midwest	137 994 (24.6)	124 (22.6)	0.68 (0.53-0.87)	
Mountain	22 694 (4.0)	26 (4.7)	0.95 (0.62-1.46)	
Pacific	76 931 (13.7)	58 (10.6)	0.71 (0.52-0.97)	
Facility type				
Community cancer program	21 108 (3.8)	4 (0.7)	1 [Reference]	<.001
Comprehensive community cancer program	198 541 (35.3)	107 (19.5)	2.48 (0.91-6.74)	
Academic or research program	229 412 (40.8)	359 (65.5)	6.26 (2.33-16.84)	
Integrated network cancer program	112 983 (20.1)	78 (14.2)	2.93 (1.07-8.05)	
Diagnosis year				
2004-2006	82 314 (14.7)	18 (3.3)	1 [Reference]	<.001
2007-2009	92 378 (16.4)	58 (10.6)	2.90 (1.71-4.93)	
2010-2012	106 930 (19.0)	62 (11.3)	2.65 (1.57-4.49)	
2013-2015	114 386 (20.4)	93 (17.0)	3.72 (2.24-6.17)	
2016-2019	166 036 (29.5)	317 (57.9)	10.18 (6.32-16.39)	
Cancer site				
Uterine	329 089 (58.6)	67 (12.2)	1 [Reference]	<.001
Ovarian	162 410 (28.9)	429 (78.3)	3.70 (2.69-5.08)	
Cervical	70 545 (12.6)	52 (9.5)	4.30 (2.76-6.70)	
Tumor stage				
I	307 100 (54.6)	33 (6.0)	1 [Reference]	<.001
II	49 628 (8.8)	35 (6.4)	3.52 (2.12-5.84)	
III	135 404 (24.1)	334 (61.0)	9.25 (6.17-13.85)	
IV	69 912 (12.4)	146 (26.6)	8.87 (5.81-13.53)	
Surgery				
No surgery	65 472 (11.7)	50 (9.1)	1 [Reference]	<.001
Any surgery	496 572 (88.4)	498 (90.9)	2.77 (1.94-3.96)	
Chemotherapy				
No	319 934 (56.9)	41 (7.5)	1 [Reference]	<.001
Yes	242 110 (43.1)	507 (92.5)	2.78 (1.93-4.02)	
Radiation				
No	413 012 (73.5)	480 (87.6)	1 [Reference]	.02
Yes	149 032 (26.5)	68 (12.4)	0.63 (0.43-0.92)	

Abbreviation: OR, odds ratio.

^a^ ORs and 95% CIs were adjusted for all variables in the table.

^b^ In the National Cancer Database, other race is not specified.

We also observed that older age at diagnosis (OR per 5-year increment, 0.89; 95% CI, 0.85-0.94) and having 2 or more comorbidities (OR, 0.56; 95% CI, 0.34-0.95) were associated with lower clinical trial enrollment odds. Area-level characteristics were related to clinical trial enrollment. Women living in zip codes with higher area-level income (quartile 4 vs quartile 1, OR, 0.65; 95% CI, 0.45-0.94) or living in zip codes with lower area-level educational attainment (quartile 4 vs quartile 1, OR, 0.41; 95% CI, 0.28-0.59) had lower odds of clinical trial enrollment. Clinical trial enrollment was lower among women living in small metropolitan (OR, 0.70; 95% CI, 0.49-0.99), medium metropolitan (OR, 0.73; 95% CI, 0.58-0.93), or urban (OR, 0.70; 95% CI, 0.51-0.96) counties but not different for women in rural counties (OR, 1.10; 95% CI, 0.55-2.16) compared with women residing in large metropolitan counties. Facility characteristics were related to clinical trial enrollment. Compared with treatment in the Northeast, those treated in the South (OR, 0.72; 95% CI, 0.57-0.90), Midwest (OR, 0.68; 95% CI, 0.53-0.87), or Pacific (OR, 0.71; 95% CI, 0.52-0.97) had lower clinical trial enrollment odds. Treatment at an academic or research program (OR, 6.26; 95% CI, 2.33-16.84) or an integrated network cancer program (OR, 2.93; 95% CI, 1.07-8.05) was associated with higher clinical trial enrollment odds compared with treatment at community cancer programs.

Over the study period, we observed higher clinical trial enrollment, with women who received a diagnosis between 2016 and 2019 being approximately 10 times more likely to be enrolled compared with those who received a diagnosis between 2004 and 2006 (OR, 10.18; 95% CI, 6.32-16.39). Patients with ovarian (OR, 3.70; 95% CI, 2.69-5.08) or cervical (OR, 4.30; 95% CI, 2.76-6.70) cancer were more likely to be enrolled in clinical trials than patients with endometrial cancer. Treatment with surgery (OR, 2.77; 95% CI, 1.94-3.96) or chemotherapy (OR, 2.78; 95% CI, 1.93-4.02) was associated with increased clinical trial enrollment odds, whereas women treated with radiation were less likely to be enrolled (OR, 0.63; 95% CI, 0.43-0.92).

PPRs according to race and ethnicity and diagnosis period and stratified by cancer site are shown in the Figure. Among patients with endometrial cancer, White and Black women were adequately represented or overrepresented (PPRs ≥1.1) in clinical trials in both time periods, with a slight decline in representation among Black women between the 2 time periods (2004-2011, PPR = 1.4; 2012-2019, PPR = 1.1). Asian and Hispanic women were inadequately represented during both time periods (PPRs ≤ 0.5). Among patients with ovarian cancer, White women were overrepresented during both time periods, whereas Asian, Black, and Hispanic women were underrepresented during both periods (PPRs ≤ 0.6). For cervical cancer, Black and White women were either overrepresented or adequately represented during both time periods, whereas Asian and Hispanic women were underrepresented. Further details of the PPRs are shown in eTable 2 in Supplement 1.

**Figure.**
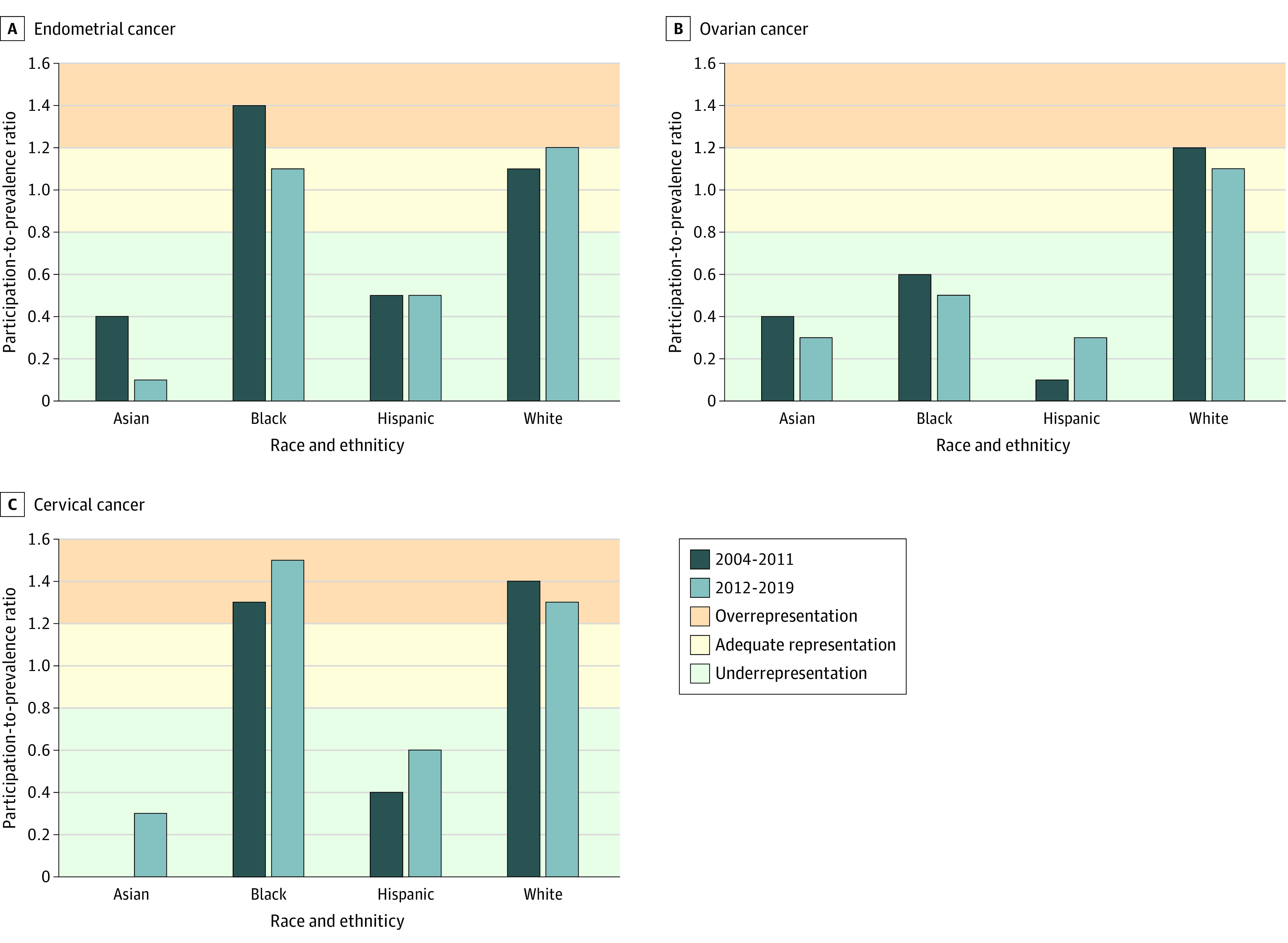
Participation-to-Prevalence Ratios for Women With Gynecologic Cancer by Diagnosis Period and Cancer Site In both time periods, Asian and Hispanic women were underrepresented in clinical trials for all 3 cancer sites. Black women with an endometrial or cervical cancer diagnosis were either adequately represented or overrepresented in both time periods, but Black women with ovarian cancer were underrepresented. White women were adequately represented or overrepresented in clinical trials for all 3 cancer sites. Numbers were too low to generate meaningful estimates for American Indian/Alaska Native, Native Hawaiian/Pacific Islander, or other race women.

## Discussion

In this retrospective cohort study of women with gynecologic cancer, we used 2 complementary approaches to evaluate racial and ethnic disparities in clinical trial enrollment. First, we examined clinical trial enrollment odds comparing participation among minoritized women with that of White women, with covariate adjustment. These analyses demonstrated lower clinical trial enrollment odds among Asian, Black, and Hispanic women compared with White women, but no difference in enrollment among American Indian/Alaska Native, Native Hawaiian/Pacific Islander, or other race women. In addition, social determinants of health, including area-level income and education, geographic region, and metropolitan status, along with certain facility characteristics, were associated with clinical trial enrollment. In the second analytic approach, analyses comparing the race-specific prevalence of clinical trial enrollment in the NCDB sample with the race-specific cancer prevalence in the US population with gynecologic cancer revealed interesting patterns. First, regardless of diagnosis period, Asian and Hispanic women with an endometrial, ovarian, or cervical cancer were underrepresented in clinical trials compared with the proportion expected on the basis of US cancer incidence. White women were either adequately represented or overrepresented for all 3 cancer sites, whereas patterns diverged for Black women: among those with endometrial or cervical cancer, adequate representation or overrepresentation was noted but among those with ovarian cancer, underrepresentation was evident. Together, these analyses provide novel information on the landscape of racial and ethnic disparities in gynecologic cancer treatment.

Prior studies^7,8^ examining clinical trial representation among patients with gynecologic cancer have compared observed case counts of racial and ethnic groups from published trials (including trials registered through ClinicalTrials.gov, Gynecologic Oncology Group–based trials,^9^ or National Cancer Institute–sponsored gynecologic cancer treatment trials^16^) to the expected racial and ethnic count obtained from population-based age-adjusted incidence rates. In support of this body of work, we identified adequate representation or overrepresentation in clinical trials among White women with endometrial, ovarian, or cervical cancers along with underrepresentation of Black patients with ovarian cancers. Our findings that Black women with endometrial or cervical cancers were adequately or overrepresented in clinical trials are in line with the findings of 2 prior studies.^8,16^ For example, Mattei and colleagues^8^ reported that Black women with either a uterine or cervical cancer were proportionately enrolled in precision medicine trials, whereas Mishkin and colleagues^16^ similarly noted no enrollment disparities for Black women with uterine or cervical cancer in National Cancer Institute–sponsored treatment trials. However, an evaluation of racial representation in Gynecologic Oncology Group–sponsored clinical trials revealed that enrollment of Black women was 9.8-fold lower than expected for endometrial cancer trials and 4.5-fold lower for cervical cancer trials.^9^ Overall, although our PPR findings indicate that Black women are being enrolled in endometrial and cervical cancer clinical trials at levels proportionate to their distribution in the population, this practice of striving for proportional enrollment is unlikely to culminate in the sample sizes needed to make well-powered conclusions about treatment efficacy within minoritized groups.^17^ Indeed, recent calls for equitable clinical trial inclusion suggest the need to recruit equal numbers of racial and ethnic groups, such that minoritized groups are overenrolled with respect to their size in the general population. A shift in this direction would allow ideally powered analyses of treatment effects within racial and ethnic groups.^18^

Disparities between Black and White populations in gynecologic oncology have been frequently investigated; however, reports focused on other racial and ethnic groups are less common. Our PPR and logistic regression analyses showing underrepresentation and lower clinical trial enrollment odds of Asian and Hispanic women agree with data published by Mattei and colleagues,^8^ where women in these groups were less commonly enrolled to precision oncology trials for ovarian and uterine cancer, with Hispanic women also less likely to be enrolled in cervical cancer clinical trials. Furthermore, in a review of National Cancer Institute–sponsored gynecologic oncology trials, Hispanic, but not Asian, women were less likely to be enrolled in ovarian, uterine, or cervical cancer clinical trials.^16^ Because of the low numbers of Native Hawaiian/Pacific Islander, American Indian/Alaska Native, and other race patients, we were unable to provide meaningful estimates of clinical trial enrollment odds or PPRs for these groups.

Apart from race and ethnicity, other factors associated with clinical trial enrollment included the presence of comorbidities, which was related to lower odds of clinical trial enrollment, in line with prior work.^19^ Although clinical trials traditionally exclude patients with medical comorbidities under the auspice of patient safety, in 2017 and 2021, the American Society for Clinical Oncology recommended broadening clinical trial eligibility to maximize generalizability.^20,21^ In addition, older age; living in zip codes with higher income; living in zip codes with lower educational attainment; living in urban, small, or medium sized counties; and treatment in the South, Midwest, and Pacific (compared with the Northeast) were associated with lower clinical trial enrollment. Treatment at an academic or research program or an integrated network cancer program was associated with higher odds of clinical trial enrollment. Most of these associations were expected on the basis of prior literature^22,23^; however, our finding that women living in areas with higher area-level income were less likely to participate in clinical trials was surprising. It is likely that area-level income also captures unmeasured neighborhood effects underlying this unexpected association. Future studies that also include individual-level income measures will be useful in contextualizing this association.

### Limitations and Strengths

Our analyses are limited by the available data within the NCDB, because we lack information on important patient and oncologic characteristics. Certain data that can affect clinical trial enrollment, including trial phase sponsor or funding source, availability of clinical trials, trial treatments (and whether they ultimately ended up becoming standard of care), and physician characteristics, are unavailable. As such, unmeasured confounding is possible in this observational study. In addition, the NCDB does not provide contextual information on the specific therapeutic area of the clinical trial (eg, cardiovascular, endocrine, or oncology); however, we assumed that an indication of clinical trial enrollment pertained to patients’ gynecologic cancer diagnosis. Moreover, we lacked details that would allow us to assess racial and ethnic differences in the pathway to clinical trial enrollment, which is important for clarifying the required intervention. For example, if racial and ethnic differences in clinical trial recommendations are apparent, technology-based interventions that screen patients and automate trial matching might be warranted.^24^ Alternatively, if we were to observe that women from minoritized racial and ethnic groups are more likely to reject clinical trials when offered, this might suggest a need for interventions aimed at the patient and physician levels to increase participation. Furthermore, because of missing values of key variables (eg, race and stage) within the NCDB data set, we excluded approximately 45% of the original sample to conduct a complete case analysis. This approach restricted the sample size, likely leading to imprecise estimates for American Indian/Alaska Native and Native Hawaiian/Pacific Islander women. It is imperative that future studies include women from these underrepresented groups to better define clinical trial disparities.

Despite these limitations, several important strengths warrant mention. First, we used a large cancer database to examine clinical trial enrollment, which is an infrequent event. Second, our analysis allowed for an internal comparison of women from different racial and ethnic groups with control for important covariates. Third, we relied on the PPR to frame representation, as opposed to age-adjusted incidence rates, which typically do not adjust for hysterectomy status, thus allowing for a potentially more accurate estimation.

## Conclusions

In this cohort study of women with gynecologic cancer, we observed that Asian, Black, and Hispanic women had lower odds of being enrolled in clinical trials, whereas women from other minoritized groups did not experience differences in clinical trial enrollment when compared with White women. Comparisons of clinical trial enrollment in this study sample with the US population revealed underrepresentation of Asian and Hispanic women with all 3 types of gynecologic cancers, underrepresentation of Black women with ovarian cancer, adequate representation of Black women with endometrial and cervical cancers, and overrepresentation of White women with all 3 gynecologic cancer types. Further work aimed at understanding the race-specific barriers and facilitators that impact enrollment of gynecologic oncology patients in clinical trials is imperative. Although we noted lower clinical trial enrollment in multiple minoritized groups, the pathways leading to these outcomes are likely diverse and will require targeted interventions.

## Notes

Supplement 1. **eTable 1.** *International Classification of Diseases for Oncology–3* Codes Including Gynecologic Cancer Histology Types **eAppendix.** Supplemental Methods **eTable 2.** Participation-to-Prevalence Ratios for Women With a Gynecologic Cancer Diagnosis According to Cancer Site and Stratified by Year of Diagnosis
eReferences

Supplement 2.
Data Sharing Statement
