# Supplementary material for: Racial and Ethnic Disparities in Clinical Trial Enrollment Among Women With Gynecologic Cancer
Source: JAMA Netw Open. 2023 Dec 7;6(12):e2346494. doi: 10.1001/jamanetworkopen.2023.46494 (PMC10704282; doi:10.1001/jamanetworkopen.2023.46494)
Supplement: Supplement 1. — eTable 1. International Classification of Diseases for Oncology–3 Codes Including Gynecologic Cancer Histology Types eAppendix. Supplemental Methods eTable 2. Participation-to-Prevalence Ratios for Women With a Gynecologic Cancer Diagnosis According to Cancer Site and Stratified by Year of Diagnosis eReferences [file jamanetwopen-e2346494-s001.pdf]

Supplemental Online Content

Khadraoui W, Meade CE, Backes FJ, Felix AS. Racial and ethnic disparities in clinical trial enrollment among women with gynecologic cancer. *JAMA Netw Open*. 2023;6(12):e2346494. doi:10.1001/jamanetworkopen.2023.46494

eTable 1. *International Classification of Diseases for Oncology–3* Codes Including Gynecologic Cancer Histology Types

eAppendix. Supplemental Methods

eTable 2. Participation-to-Prevalence Ratios for Women With a Gynecologic Cancer Diagnosis According to Cancer Site and Stratified by Year of Diagnosis

eReferences

This supplemental material has been provided by the authors to give readers additional information about their work.

© 2023 Khadraoui W et al. *JAMA Network Open.*

eTable 1. *International Classification of Diseases for Oncology–3* Codes Including Gynecologic Cancer Histology Types		
**Cancer site**	**Histology**	**Codes**
Uterine (C54.0-C54.9, C55.9)	Endometrioid adenocarcinoma	8140, 8210, 8211, 8260, 8261, 8262, 8263, 8560, 8570, 8380, 8381, 8382, 8383
	Mucinous	8480, 8481, 8482
	Serous	8441, 8460, 8461
	Carcinosarcoma	8950, 8951, 8980, 8981, 8982
	Clear cell	8310
	Mixed cell	8323, 8255
Cervical (C53.0, 53.1, 53.8, 53.9)	Squamous cell carcinoma	8070, 8071, 8072, 8076
	Adenocarcinoma	8140
Ovarian (C56.9)	Serous	8441, 8460, 8461
	Endometrioid	8380, 8381, 8382, 8383, 8560, 8570, 8950, 8951, 8980
	Clear cell	8005, 8310, 8313
	Mucinous	8470, 8471, 8480, 8481
	Other epithelial	8010, 8050, 8140, 8255, 8260, 8323
Fallopian tube (C57.0)	Serous	8441, 8460, 8461
	Endometrioid	8380, 8381, 8382, 8383, 8560, 8570, 8950, 8951, 8980
	Clear cell	8005, 8310, 8313
	Mucinous	8470, 8471, 8480, 8481
	Other epithelial	8010, 8050, 8140, 8255, 8260, 8323
Peritoneum (C48.1, C48.2)	Serous	8441, 8460, 8461
	Endometrioid	8380, 8381, 8382, 8383, 8560, 8570, 8950, 8951, 8980
	Clear cell	8005, 8310, 8313
	Mucinous	8470, 8471, 8480, 8481
	Other epithelial	8010, 8050, 8140, 8255, 8260, 8323
Retroperitoneum (C48.0)	Serous	8441, 8460, 8461
	Endometrioid	8380, 8381, 8382, 8383, 8560, 8570, 8950, 8951, 8980
	Clear cell	8005, 8310, 8313
	Mucinous	8470, 8471, 8480, 8481

© 2023 Khadraoui W et al. *JAMA Network Open.*

**eTable 1.** ***International Classification of Diseases for Oncology–3*** **Codes Including Gynecologic Cancer Histology Types**		
**Cancer site**	**Histology**	**Codes**
	Other epithelial	8010, 8050, 8140, 8255, 8260, 8323

© 2023 Khadraoui W et al. *JAMA Network Open.*

eAppendix. Supplemental Methods

Race

In the National Cancer Database (NCDB), race is coded as White; Black; American Indian, Aleutian, or Eskimo; Chinese; Japanese; Filipino; Hawaiian; Korean; Vietnamese; Laotian; Hmong; Kampuchean (including Khmer and Cambodian); Thai; Asian Indian or Pakistani, not otherwise specified (NOS); Asian Indian; Pakistani; Micronesian, NOS; Chamorran; Guamanian, NOS; Polynesian, NOS; Tahitian; Samoan; Tongan; Melanesian, NOS; Fiji Islander; New Guinean; Other Asian, including Asian, NOS and Oriental, NOS; Pacific Islander, NOS; Other; and unknown (excluded from this analysis). We created the following race categories based on the Office of Management and Budget standards: 1. White; 2. Black; 3. Asian [Japanese; Filipino; Korean; Vietnamese; Laotian; Hmong; Kampuchean (including Khmer and Cambodian); Thai; Asian Indian or Pakistani, NOS; Asian Indian; Pakistani; Other Asian, including Asian, NOS and Oriental, NOS]; 4. American Indian/Alaska Native (American Indian, Aleutian, or Eskimo); 5. Native Hawaiian/Pacific Islander (Hawaiian; Micronesian, NOS; Chamorran; Guamanian, NOS; Polynesian, NOS; Tahitian; Samoan; Tongan; Melanesian, NOS; Fiji Islander; New Guinean; Pacific Islander, NOS), and 6. Other.

Area-level income

Area-level income "for each patient's area of residence is estimated by matching the zip code of the patient recorded at the time of diagnosis against files derived from the 2020 American Community Survey data, spanning years 2016-2020 and adjusted for 2020 inflation. Household income is categorized as quartiles based on equally proportioned income ranges among all US zip codes."

Categories
1. < $46,277
2. $46,277 - $57,856
3. $57,857 - $74,062
4. $74,063 +

Area-level education

Area-level education "for each patient's area of residence is estimated by matching the zip code of the patient recorded at the time of diagnosis against files derived from the 2020 American Community Survey data, spanning years 2016-2020. This item provides a measure of the number of adults age 25 or older in the patient's zip code who did not graduate from high school, and is categorized as equally proportioned quartiles among all US zip codes."

Categories
1. 15.3% +
2. 9.1%-15.2%
3. 5.0%-9.0%
4. <5.0%

Metro status

In the NCDB, rural-urban continuum codes "form a classification scheme that distinguishes metropolitan (metro) counties by the population size of their metro area, and nonmetropolitan (non-metro) counties by degree of urbanization and adjacency to a metro area or areas. The metro and non-metro categories have been subdivided into three metro and six non-metro groupings, resulting in a nine part county codification."

Categories
1. Counties in metro areas of 1 million population or more;
2. Counties in metro areas of 250,000 to 1 million population;
3. Counties in metro areas of fewer than 250,000 population;
4. Urban population of 20,000 or more, adjacent to a metro area;
5. Urban population of 20,000 or more, not adjacent to a metro area;

© 2023 Khadraoui W et al. *JAMA Network Open.*

6. Urban population of 2,500 to 19,999, adjacent to a metro area;
7. Urban population of 2,500 to 19,999, not adjacent to a metro area;
8. Completely rural or less than 2,500 urban population, adjacent to a metro area;
9. Completely rural or less than 2,500 urban population, not adjacent to a metro area

Based on previous literature[1], we collapsed categories to define metro status as 5 groups: 1. Large metropolitan (metro) county (population > 1 million); 2. Medium metro country (population 250,000-1 million); 3. Small metro country (population <250,000); 4. Urban; and 5. Rural. The urban category contains the following: urban population of 20,000 or more, adjacent to a metro area; urban population of 20,000 or more, not adjacent to a metro area; urban population of 2,500 to 19,999, adjacent to a metro area; and urban population of 2,500 to 19,999, not adjacent to a metro area. The rural category contains the following: completely rural or less than 2,500 urban population, adjacent to a metro area; and completely rural or less than 2,500 urban population, not adjacent to a metro area.

Stage
We categorized stage (I, II, III, IV) using the American Joint Commission on Cancer pathological and/or clinical staging criteria. Based on clinical practice, we prioritized pathologic staging criteria for all cancer sites. For cervical or ovarian cancer patients with missing pathologic stage, we supplemented with clinical stage.

Cancer site-specific exclusions
Additional cancer site-specific exclusions were as follows: endometrial cancer (unknown if surgery performed, no surgery performed, ungraded endometrioid histology); cervical cancer (surgery types of local tumor destruction: photodynamic therapy or local tumor destruction: thermal ablation); ovarian cancer (ungraded endometrioid histology, ungraded serous histology); peritoneal cancer (ungraded endometrioid histology, ungraded serous histology, surgery types of local tumor destruction, NOS, local tumor destruction: laser, any combination of local tumor excision, polypectomy, or excisional biopsy with cryosurgery, any combination of local tumor excision, polypectomy, or excisional biopsy with laser ablation, or local tumor excision: polypectomy); retroperitoneum (ungraded endometrioid histology, ungraded serous histology); and fallopian tube (ungraded endometrioid histology, ungraded serous histology, surgery types of any combination of local tumor excision, polypectomy, or excisional biopsy with electrocautery, any combination of local tumor excision, polypectomy, or excisional biopsy with laser ablation, or local tumor excision: polypectomy).

Calculation of the participation to prevalence ratios (PPR)
In the SEER*stat frequency session we applied the following restrictions: malignant behavior, first matching record for each person, women aged ≥ 18 years, diagnosed between 2004 and 2019 with an endometrial, ovarian (plus fallopian tube, retroperitoneal, and peritoneal), or cervical cancer with the histology types identified in **eTable 1**.[2] Because we could not apply the exact exclusion criteria used in the NCDB analysis to the Surveillance, Epidemiology, and End Results Program (SEER) population due to the absence of certain variables within SEER (*e.g.,* facility location, facility type, income, education, etc.), we instead present the race/ethnic distribution of NCDB clinical trial enrollment among women aged ≥ 18 years, diagnosed between 2004 and 2019, with an endometrial, ovarian (plus fallopian tube, retroperitoneal, and peritoneal), or cervical cancer with the histology types identified in **eTable 1**.This allowed equivalent denominators for both frequencies.

© 2023 Khadraoui W et al. *JAMA Network Open.*

eTable 2. Participation-to-Prevalence Ratios for Women With a Gynecologic Cancer Diagnosis According to Cancer Site and Stratified by Year of Diagnosis									
	**Endometrial cancer**			**Ovarian cancer**			**Cervical cancer**		
	**2004-2011**								
	Clinical trial enrollment %[a]	Cancer prevalence %[b]	PPR[c]	Clinical trial enrollment %[a]	Cancer prevalence %[b]	PPR[c]	Clinical trial enrollment %[a]	Cancer prevalence %[b]	PPR[c]
Asian	2.8	6.6	0.4	2.4	6.8	0.4	0.0	8.1	0.0
Black	11.1	8.1	1.4	4.2	6.7	0.6	20.0	14.9	1.3
Hispanic	5.6	10.5	0.5	0.6	10.9	0.1	10.0	23.9	0.4
White	80.6	72.7	1.1	92.8	74.4	1.2	70.0	51.0	1.4
	**2012-2019**								
	Clinical trial enrollment %[a]	Cancer prevalence %[b]	PPR[c]	Clinical trial enrollment %[a]	Cancer prevalence %[b]	PPR[c]	Clinical trial enrollment %[a]	Cancer prevalence %[b]	PPR[c]
Asian	1.1	7.9	0.1	2.3	8.9	0.3	3.0	9.0	0.3
Black	11.0	9.9	1.1	3.5	7.6	0.5	22.4	14.7	1.5
Hispanic	7.7	14.2	0.5	3.7	14.4	0.3	14.9	24.2	0.6
White	79.1	65.2	1.2	88.7	67.5	1.3	58.2	49.3	1.2

a From the National Cancer Database

b From the Surveillance, Epidemiology, End Results Program (Incidence - SEER Research Data, 17 Registries (Alaska Native Tumor Registry, Connecticut, Atlanta, Greater Georgia, Rural Georgia, San Francisco-Oakland, San Jose-Monterey, Greater California, Hawaii, Iowa, Kentucky, Los Angeles, Louisiana, New Mexico, New Jersey, Seattle-Puget Sound, Utah), Nov 2022 Sub (2000-2020))

c clinical trial enrollment %/cancer prevalence %

© 2023 Khadraoui W et al. *JAMA Network Open.*

eReferences

1. Obeng-Gyasi S, Timsina L, Bhattacharyya O, Fisher CS, Haggstrom DA. Breast Cancer Presentation, Surgical Management and Mortality Across the Rural-Urban Continuum in the National Cancer Database. *Annals of surgical oncology.* 2020;27(6):1805-1815.
2. Surveillance Research Program, National Cancer Institute SEER*Stat software (www.seer.cancer.gov/seerstat) version 8.4.1. Accessed September, 2015.

© 2023 Khadraoui W et al. *JAMA Network Open.*
