# Supplementary material for: Racial and Ethnic Disparities in Clinical Trial Enrollment Among Women With Gynecologic Cancer
Source: JAMA Netw Open. 2023 Dec 7;6(12):e2346494. doi: 10.1001/jamanetworkopen.2023.46494 (PMC10704282; doi:10.1001/jamanetworkopen.2023.46494)
Supplement: Supplement 2. — Data Sharing Statement [file jamanetwopen-e2346494-s002.pdf]

Data Sharing Statement

Khadraoui. Racial and Ethnic Disparities in Clinical Trial Enrollment Among Women With Gynecologic Cancer. *JAMA Netw Open*. Published December 07, 2023.
doi:10.1001/jamanetworkopen.2023.46494

Data
Data available: No

Additional Information
Explanation for why data not available: The data for this study were acquired by application to the restricted access National Cancer Database and the data use agreement does not allow data sharing
